# ZnO Nanowires: Growth, Properties, and Energy Applications

**DOI:** 10.3390/nano13182519

**Published:** 2023-09-08

**Authors:** Vincent Consonni

**Affiliations:** Université Grenoble Alpes, CNRS, Grenoble INP, LMGP, F-38016 Grenoble, France; vincent.consonni@grenoble-inp.fr

As a biocompatible semiconductor composed of abundant elements, ZnO, in the form of nanowires, exhibits remarkable properties, mainly originating from its wurtzite structure and correlated with its high aspect ratio at nanoscale dimensions. ZnO nanowires have thus received increasing interest in the community and have specifically emerged as a potential building block for a wide variety of devices in the field of energy conversion. Among the different energy conversion applications, ZnO nanowires have, to name just two examples, been integrated into nanostructured solar cells and piezoelectric devices. Despite the vast number of publications in the field, there is still a significant need to explore the growth of ZnO nanowires, to more precisely elucidate and control their fundamental properties, and to improve their integration into real-world engineering devices.

In terms of growth, one of the most difficult challenges consists in integrating ZnO nanowires over dedicated localized areas and dedicated substrates, both of these being relevant for the targeted devices. Durbach et al. report a nano-second laser irradiation process for generating Au catalysts over dedicated areas, thus controlling the position of ZnO nanowires grown by chemical vapor deposition through a selective growth approach compatible with large surfaces [1]. Schaper et al. show the formation of ZnO nanowires over single-walled carbon nanotubes and graphene using a full chemical vapor deposition approach, further achieving selective growth over dedicated areas [2]. Another challenge consists in developing innovative heterostructures made of ZnO nanowires combined with a selected semiconductor. Jin et al. develop the growth of semiconducting shells (i.e., ZnS and Ag_2_S) deposited by successive ionic layer adsorption and reaction on ZnO nanowires, further revealing their UV-sensing properties [3]. Zhang et al. report on the fabrication of heterostructures made from ZnO nanorods covered with TiO_2−x_ mesoporous spheres, revealing their properties for photocatalytic hydrogen production [4].

The integration of ZnO nanowires into nanostructured solar cells as an electron-transporting material is driven by the expected benefits of light-trapping phenomena and efficient charge carrier management. However, beyond the proof-concept of ZnO nanowire-based solar cells, the need to carefully optimize the dimensional parameters suffers from technological challenges. Sekar et al. report on the optimization of the dimensions of ZnO nanowires and their impact on the photovoltaic properties of FACsPb(IBr)_3_ perovskite solar cells, further exploring the use of a carbazole-based hole-transporting material [5]. Hector et al. investigate the effect of the thickness of the Sb_2_S_3_ shell over the photovoltaic properties of extremely thin absorber solar cells, revealing the dimensional trade-off required [6].

ZnO nanowires, with their growth direction oriented along the piezoelectric and polar *c*-axis, act as the active layer in piezoelectric devices, which are largely developed using a vertically integrated configuration. By combining finite-element method calculations with experimental data available in the literature, Lopez-Garcia et al. report on dimensional roadmap and optimization guidelines, showing that the range of optimal radius, that fully deplete ZnO nanowires in terms of charge carriers, depends on the growth technique [7]. Lopez-Garcia et al. reveal the fabrication of gravure-printed ZnO seed layers as an alternative process in order to subsequently form ZnO nanowires over flexible polymer substrates, further characterizing their piezoelectric properties using piezoresponse force microscopy [8]. Tlemcani et al. show the integration of ZnO nanowires into flexible piezoelectric nanogenerators and compare their performance using two seed layer structures (i.e., Au/ZnO vs. ITO/ZnO) [9]. Zhai et al. report on the combination of ZnO nanowires with a cellulose nanofiber film, further revealing their electromechanical and UV-sensing properties [10].

Ultimately, assembling ZnO nanowires into hierarchical structures represents a promising approach for further increasing their integration into engineering devices. Di Mari et al. report on the formation of ZnO nanostars made of agglomerated nanowires and explore their properties as pseudo-capacitors for energy storage [11].

In summary, this Special Issue brings together more than 80 authors from different countries, who submitted 11 original research articles conveying their foundational research dedicated to ZnO nanowires. Overall, if the present Special Issue cannot fully reflect the high diversity rapidly developing in the community of ZnO nanowires, it will certainly contribute to research interest in the field.
